# Safety and efficacy of substance-based medical devices: Design of an *in vitro* barrier effect test

**DOI:** 10.3389/fdsfr.2023.1124873

**Published:** 2023-04-11

**Authors:** Rebecca Bassetto, Stefano Perin, Emanuele Amadio, Samuele Zanatta, Davide Nenzioni, Walter Bertin

**Affiliations:** Labomar S.p.a, Istrana, Italy

**Keywords:** medical devices, franz cell, biomimetic membrane, permeability, barrier effect, nutraceuticals

## Abstract

This study aims to develop an *in vitro* barrier effect test over biomimetic membrane, which is useful to establish the film forming ability of a substance-based medical device (SB-MD). The method contemplates a multiparametric approach including: *i*) the measurement of the transmembrane passage of a molecular-like marker over a lipid-impregnated biomimetic membrane (simulating the skin and gastro-intestinal and buccal tissues) by using a static diffusion cell apparatus (Franz cell); and *ii*) the evaluation of the integrity of the membrane (colorimetric test). In the first step, a series of lipid-impregnated biomimetic membranes (simulating gastro-intestinal, buccal, and skin tissues) were implemented and their permeability performance validated using model drugs (caffeine and acyclovir) by referring to literature data. As a result, the apparent permeability (P_app_) of caffeine over the biomimetic gastro-intestinal membrane (P_app_ = 30.5E-6 cm/s) was roughly comparable to the literature values obtained with Caco-2 cell line membrane (P_app_ = 30.8E-6 cm/s) and with the Franz cell method (P_app_ = 36.2E-6 cm/s). Acyclovir was shown to be a poorly permeable substance both in the literature and experimental data. Following this step, the permeability study was extended to both biomimetic buccal and skin (STRAT-M^®^) membranes: for caffeine, biomimetic gastro-intestinal membrane was the most permeable (P_app_ = 30.5E-6 cm/s), followed by the buccal (P_app_ = 18.2E-6 cm/s) then the skin (P_app_ = 0.5E-6 cm/s) biomimetic membranes. In a second part of the work, the barrier effect test was developed following a similar permeability-like approach. The protocol was designed with the idea of assessing the capacity of a certain product to prevent the passage of caffeine across the biomimetic membrane with respect to a negative and positive control. The untreated membrane was the negative control, while membrane covered with a Vaseline film was the positive. As a last step, the developed barrier effect protocol was applied to an experimental gel-like SB-MD under development for the treatment of aphthae (Aphthae gel, an invented trade name), herein used as a case study. Regarding the results, Aphthae gel reduced the caffeine passage by 60.3%, thus highlighting its effectiveness to form a protective film. Overall, these results provide important knowledge and may pave the way for the use—including for industrial applications—of these simple but effective biomimetic membranes for carrying out high throughput screening necessary to design safe and effective SB-MDs before proceeding further with clinical trials, as requested by the regulations.

## 1 Introduction

In response to the new European Regulations for substance-based medical devices (SB-MD), there is the necessity to deeply study their safety and efficacy and thus to develop new experimental protocols to demonstrate the concept of their non-pharmacological mechanisms of action (UE, 2021; Giovagnoni, 2022). By definition, “*substance-based medical devices are medical devices that are composed of substances or combinations of substances that are intended to be introduced into the human body via a body orifice or applied to the skin and that are absorbed by or locally dispersed in the human body*”. Although they are herbal-like medicinal products in their presentation and pharmaceutical form, they achieve their principal intended effect *via* a physicochemical and/or physical mechanism of action (including mechanical action, a physical barrier such as a film, lubrication, hydration or dehydration, and pH modification) (Fimognari et al., 2022; Manellari et al., 2022). The ISO 10993 sets a series of standards and guidance for the biological evaluation of medical devices within a risk management process as part of the overall evaluation and development of the medical device (ISO, 2020). In this context, the ISO 10993-2 describes animal welfare aspects regarding the performing of animal studies for the biological evaluation of medical devices, thereby also emphasizing the 3Rs: the replacement, reduction, and refinement of animal studies. ISO 10993-1,-2, and −23 promote the use of *in vitro* tests instead of *in vivo* to support animal welfare, saying that “*in vitro tests have preference over in vivo tests when appropriately validated and providing equally relevant information to that obtained from in vivo tests*”. Despite these standards, to date there is no regulatory reference explaining in detail the operational procedures needed to evaluate the safety and effectiveness of SB-MDs.

Therefore, in this study, the barrier effect method was developed using an *in vitro* animal-free biomimetic approach to evaluate the film-forming ability of SB-MDs as a part of the experimental process necessary to establish the safety and effectiveness of SB-MD products. The barrier effect is necessary to measure the ability of a given device to protect human tissues from external agents by promoting the maintenance of its normal physical-chemical balance. From a technical point of view, the herein proposed barrier effect assay takes advantage of the permeability study test, in which caffeine was selected as a probe to assess the propensity of a given product to form a protective film due to its ability to permeate different models of human tissue even in the absence of damage. Moreover, caffeine is the chemical reference for *in vitro* absorption studies as stated by the OECD 428 and related Guidance Documents (OECD, 2004). In general, a permeability assay measures the flux and the kinetic profile of a defined substance from a donor into an acceptor compartment through the respective membrane (di Cagno et al., 2015). The kinetic profile reflects the changes in drug concentration over time and diffusion through the membrane. The permeability coefficient calculated out of this study determines the rate of migration of a substance through the membrane. For this purpose, several well-characterized *in vitro* permeability prediction methods have been developed in recent decades (Corti et al., 2006a; Corti et al., 2006b). Moreover, many organizations [i.e., the Organization for Economic Co-operation and Development (OECD), the United States Environmental Protection Agency, and the European Commission Scientific Committee on Consumer Products (SCCP)] have produced extensive guidelines to assist companies and organizations towards the implementation of harmonized *in vivo* and/or *in vitro* absorption studies (OECD, 2004; SCCP, 2010; Hopf et al., 2020). Regarding absorption, the above-described guidelines for the *in vitro* methodologies outline the following criteria: *i*) the use of static diffusion cell apparatus; *ii*) the use of an appropriate membrane positioned between the upper and lower chambers of a static diffusion cell; *iii*) the test sample should remain in contact with the membrane on the donor side for a defined period (from 0.5 h up to 24 h); *iv*) the receptor fluid may be a degassed saline or buffered saline solutions having a physiological pH and temperature; *v*) the receptor fluid should be sampled to obtain an absorption-time profile by quantifying a defined marker compound *via*, for example, high-performance liquid chromatography (HPLC) and/or UV-Vis spectroscopies; and *vi*) at the end of the experiment, the integrity of the membrane should be checked by evaluating the penetration of a marker molecule. In general, however, these indications are intended to be modulable and any deviation from this principle is possible when justified by the study case.

Accordingly with those publications and guidelines, herein, the Franz cells system was used as a static diffusion cell. This apparatus has been widely used to study the *in vitro* permeation of pharmaceutical, nutraceutical, or topical products thanks to its simplicity, reproducibility, and cost-effectiveness (Ng et al., 2010; Casiraghi et al., 2017; Salamanca et al., 2018). Moreover, the system was previously validated by Texeira et al., who studied the intestinal permeability of BCS model drugs over biomimetic intestinal membranes, comparing the data with Caco-2 cells (Teixeira et al., 2020).

Focusing on the membrane, many different human (e.g., human cadaver, surgical biopsies, and skin from cosmetic surgeries) or animal (e.g., pig, rodent) tissues can be in principle used to carry out a permeability test. However, the use of biological tissue has several drawbacks, including ethical issues, difficult and time-consuming preparation, handling, and maintenance of freshly excised tissues, the possibility of tissue damages, and high sample to sample biological variability even within the same species (e.g., depending on age, sex, race), with consequently poor reproducibility in permeation results and lack of full resemblance with *in vivo* data. For these reasons, in recent years, artificial biomimetic membranes have progressively gained interest as an alternative model to *in vivo* applications. Moreover, several studies have been published demonstrating a good relationship between the permeability data for transcellularly transported drugs measured using synthetic membranes and those obtained with cell-based model tissue (Corti et al., 2006a; Corti et al., 2006b; Haq et al., 2018a; Berben et al., 2018; Haq et al., 2018b; Mura et al., 2018; Teixeira et al., 2020; Fedi et al., 2021). It is worth noting this biomimetic membrane may be correctly predicting only the passive transcellular absorption because these artificial membranes do not have any transporters. However, since most commercial drugs (80%–95%) are primarily absorbed by passive diffusion (Loftsson et al., 2006; Di et al., 2012), the use of artificial membranes offers an effective high throughput approach for the drug absorption and represents a very useful tool for the early stages of pre-clinical studies. Moreover, synthetic membranes are also preferred as they are more easily resourced, less expensive, and structurally simpler than real tissue. Furthermore, they exhibit superior permeation data reproducibility as *in vivo* variables are eliminated.

From a structural point of view, the artificial biomimetic membrane is a multi-component system formed by a porous polymeric support with a very tightly packed lipidic-like surface layer that creates a defined organization at the molecular level resembling both the morphology and the lipophilic properties observed in the desired human biological barrier. Generally, the superficial lipidic film is composed of an oleic mixture of phospholipids and sterols (Corti et al., 2006a; Corti et al., 2006b; Eeman and Deleu, 2010; Khdair et al., 2013; Mura et al., 2018). As recently separately reported by Corti et al., Mura et al., and Khdair et al., biomimetic artificial membranes may be efficiently produced starting from different dialysis membranes opportunely impregnated with a mixture of n-octanol, Lipoid^®^E80, and cholesterol (Corti et al., 2006a; Corti et al., 2006b; Khdair et al., 2013; Mura et al., 2018). In their publications, the authors systematically tested a series of polymeric filters with different structural and chemical natures (e.g., type of polymer, pore size, percent of porosity, and thickness) impregnated with a diverse ratio of the lipidic mixtures. The purpose of these studies was to reproduce these artificial membranes and use them to predict drug absorption in human gastro-intestinal and buccal tissues.

In addition to intestinal and oral absorption, dermal absorption assays are used to predict risks from the exposure to chemicals as well as to demonstrate the efficacy of cosmetics, medical devices, and of some topical-delivery therapeutic active ingredients. In the past, the most used dermal tissue was “*ex-vivo*” porcine skin, despite its lower barrier function compared with human skin. Nowadays, also for ethical reasons, the use of animal tissues has been restricted and, thus, numerous skin surrogate systems and human skin equivalents (HSEs) have been developed (Pellegatta et al., 2020). In this context, Strat-M^®^ is the most used synthetic non-animal-based membrane model for transdermal diffusion tests. This membrane is a multi-layered polyether sulphone support specially designed to mimic the skin structure (e.g., stratum corneum, dermis, and subcutaneous tissue) and covered with skin lipids (e.g., ceramides, cholesterol, and free fatty acids). The hydrophobic lipidic mixture coated on the membrane is composed of the main stratum corneum lipids. The polyether sulfone cut-off has been designed to mimic the human skin morphology more closely than other artificial membranes. These physio-chemical properties make the Strat-M^®^ membrane an interesting and recommended model alternative to evaluate the skin permeability of molecules. Moreover, many studies have shown that Strat-M^®^ membrane can be used as a surrogate for human skin to study the diffusion characteristics of a wide range of compounds for topical and transdermal formulations, providing close transport correlation characteristics to human skin (Haq et al., 2018a; Haq et al., 2018b).

With this work, we demonstrate that it may be possible to exploit the use of these simple and effective biomimetic membranes for developing a barrier effect test. The results of this study show that biomimetic membranes represent a useful tool for the preliminary high throughput screening of film forming formulation candidates to be further tested for their efficacy and safety in clinical trials, as requested by the regulations related to SB-MDs.

The protocols here proposed were adequately designed to be suitable for industrial use, for which having an experimental high throughput screening is fundamental to quickly creating safe and effective formulations. As a case study, the method was then applied to an experimental gel-like SB-MD under development for the treatment of aphthae (Aphthae gel, an invented trade name). This SB-MD was designed for the treatment of aphthae, stomatitis, and microlesions of the mouth. It forms a protective film on microlesions that, thanks to the effectiveness of selected natural extracts, reduces painful symptoms and burns and promotes re-epithelialization phenomena.

## 2 Materials and methods

### 2.1 Materials

Polymeric dialysis-like supports were purchased from Millipore^®^ (Mixed Cellulose Esters VCWP02500, 0.1 μm × 25 mm, white plain; Mixed Cellulose Esters VSWP02500, 0.025 μm × 25 mm, white plain; New York, NY, United States). The lipid phase used for the impregnation of the porous supports consisted of Lipoid^®^ E80 by Lipoid (Ludwigshafen, Germany), and cholesterol and *n*-octanol purchased from Sigma–Aldrich (Milan, Italy). Caffeine and acyclovir reference standards were purchased from Merck (Darmstadt, Germany). Water (HPLC grade), methanol (HPLC grade) and acetonitrile (HPLC grade) were purchased from Carlo Erba Reagents (Cornaredo, Milan, Italy). All reagents were used without further purification. Aphthae gel was provided from Labomar (batch K1861 T, exp 2024/07).

#### 2.1.1 Instrument and chromatographic conditions

The standard stock solutions quantification was performed using a UV-1280 spectrophotometer (Shimadzu, Japan). The HPLC-UV analyses were performed on VANQUISH Core/Ultimate 3,000 from Thermo Fisher Scientific (Waltham, Massachusetts, United States) which included a pump, autosampler, column oven, and diode array detector (DAD). The reverse phase column Acclaim™ C18 (150 mm × 4.6 mm, 5 mm particle size) from Thermo Fisher Scientific (Waltham, United States) was used and maintained at 20°C. The injection volume of standards and samples were 10 μL for caffeine standard solutions and 20 μL for acyclovir standard solutions. The detector wavelength was set at 275 nm for caffeine and 254 nm for acyclovir. The mobile phase for the methods consisted of A: water, B: acetonitrile and C: methanol. The analytical methods for caffeine and acyclovir were validated using the elution gradients reported in Supplementary Tables S13, S14. The data were acquired with a Chromeleon 7 (Thermo Fisher Scientific, Waltham, Massachusetts, United States) and processed using Microsoft Excel (Microsoft, Redmond, Washington, United States).

### 2.2 Methods

#### 2.2.1 Sample preparation for HPLC analysis

##### 2.2.1.1 Stock and standard solutions

Stock solutions were prepared by weighing 50 mg of caffeine reference standard and 25 mg of acyclovir reference standard into 50 mL (MeOH 20% *v/v* in water) and 20 mL (ACN 2% *v/v* in water) volumetric flasks, respectively. The standard solutions were stirred for 1 h and the concentrations were checked *via* UV-vis spectrophotometry analysis. For the caffeine stock solution, 963 μg/mL was derived and 1,180 μg/mL was derived for the acyclovir stock solution. The caffeine standard solutions were prepared by diluting a specific amount of stock solution in the solvent (MeOH 20% *v/v* in water) to obtain a range of concentrations: 0.94 μg/mL, 4.7 μg/mL, 9.6 μg/mL, 24.0 μg/mL, 48.2 μg/mL, 77.0 μg/mL, 93.5 μg/mL, 115.6 μg/mL, and 192.6 μg/mL. Moreover, for LOD and LOQ determination, concentrations of 0.03 μg/mL and 0.05 μg/mL were prepared, respectively. Acyclovir standard solutions were prepared by diluting a specific amount of stock solution in the solvent (ACN 2% *v/v* in water) to obtain a range of concentrations: 0.1 μg/mL, 0.5 μg/mL, 1 μg/mL, 5 μg/mL, 10 μg/mL, 25 μg/mL, and 50 μg/mL. For acyclovir LOD and LOQ determination, concentrations of 0.03 μg/mL and 0.05 μg/mL were prepared, respectively. Each standard solution was filtered through a 0.20 μm syringe filter.

##### 2.2.1.2 Specificity

The samples for specificity evaluation consisted of: placebo solutions (PBS buffer solutions without caffeine or acyclovir) and PBS solutions spiked with 100% of analyte. PBS buffer was prepared as follows: NaCl 8.00 g/L, KCl 0.200 g/L, Na_2_HPO_4_∙2H_2_O 1.44 g/L, and KH_2_PO_4_ 0.245 g/L were dissolved in water and pH was adjusted to 7.4. The placebo sample for caffeine was prepared by diluting PBS buffer in MeOH 20% *v/v* in water (1:10), and the placebo sample for acyclovir was prepared by diluting PBS buffer in ACN 2% *v/v* in water (1:10). Spiked sample solutions were prepared by weighing 10 mg of caffeine reference standard and 1 mg of acyclovir reference standard into 10 mL volumetric flasks and solubilized in PBS buffer. Dilutions of 1:10 were used in MeOH 20% *v/v* in water for caffeine and ACN 2% *v/v* in water for acyclovir. For the Aphthae gel case study, the samples consisted of: placebo solutions with 200 mg of Aphthae gel in PBS buffer (without caffeine or acyclovir) and spike solutions with 200 mg of Aphthae gel in PBS buffer with spike 100% analyte addition (with caffeine or acyclovir). Caffeine and acyclovir placebo samples were prepared by weighing 200 mg of Aphthae gel and dissolving in 10 mL volumetric flasks containing PBS buffer. The solution was stirred for 1 h at room temperature. Before injection, a 1:10 dilution in MeOH 20% *v/v* in water was used for caffeine, and the placebo sample for acyclovir was prepared by diluting Aphthae gel PBS buffer solution in ACN 2% *v/v* in water (1:10). Additionally, spiked sample solutions were prepared by weighing 10 mg of caffeine reference standard and 1 mg of acyclovir reference standard into two different 10 mL volumetric flasks with 200 mg of Aphthae gel and solubilized in PBS buffer. The solutions were stirred for 1 h at room temperature. Dilutions of 1:10 were used in MeOH 20% *v/v* in water for Caffeine and ACN 2% *v/v* in water for acyclovir. Each sample was filtered through a 0.20 µm syringe filter and single injection was performed.

##### 2.2.1.3 Precision and accuracy

Caffeine spiked sample solutions were prepared by weighing 8, 10, and 12 mg of caffeine reference standard into 10 mL volumetric flasks and solubilized in PBS buffer. The same was done for acyclovir spiked sample solutions, by weighing 0.8, 1, and 1.2 mg of acyclovir reference standard. Dilutions of 1:10 were used in MeOH 20% *v/v* in water for caffeine and ACN 2% *v/v* in water for acyclovir. For the Aphthae gel case study, caffeine spiked sample solutions were prepared by weighing 8, 10, and 12 mg of caffeine reference standard into 10 mL volumetric flasks with 200 mg of Aphthae gel and solubilized in PBS buffer. The solutions were stirred for 1 h at room temperature. The same was done for acyclovir spiked sample solutions, by weighing 0.8, 1, and 1.2 mg of acyclovir reference standard. Dilutions of 1:10 were used in MeOH 20% *v/v* in water for caffeine and ACN 2% *v/v* in water for acyclovir. Each sample was filtered through a 0.20 μm syringe filter and triplicate injection was performed.

#### 2.2.2 Preparation of biomimetic membrane

The membrane’s support was functionalized by immersion in a lipid mixture solution composed of phospholipids (Lipoid^®^ E80), cholesterol, and n-octanol for 60 min at room temperature. Briefly, the lipid phase solution for the preparation of intestinal membranes was a mixture of 1.7% phospholipids (Lipoid^®^ E80, Ludwigshafen, Germany), 2.1% cholesterol (Sigma–Aldrich Chemical Co., Milan, Italy), and 96.2% *n*-octanol (Sigma–Aldrich Chemical Co., Milan, Italy); for the preparation of buccal biomimetic membrane, the lipid phase solution was composed of 3.3% Lipoid E80, 3.2% cholesterol, and 93.5% *n*-octanol. Excess lipids were absorbed with filter paper over 30 min. Next, all impregnated membranes were weighed, evaluated to check for accuracy (intestinal membranes: 50% ± 5; buccal 41% ± 2), and then stored in a freezer for at least 24 h for stabilization.

#### 2.2.3 Permeability studies

Permeability studies were performed with a Franz cell (Copley Scientific, United Kingdom), studying the permeability of specific compounds through the membrane. Impregnated artificial membranes were positioned between upper and lower part of the diffusion cells. The receiving chamber (10.5 mL) was filled with degassed phosphate-buffered solution (PBS), pH 7.4 (USP 32), left under stirring (200 rpm) and the temperature was kept constant (37.0°C ± 0.5°C). In the donor, 1 mL of drug (caffeine 10 mg/mL, acyclovir 1 mg/mL) was added and covered to prevent evaporation. Samples from the receiving chamber were collected from 0 up to: 4 h for intestinal membranes, 3 h for buccal membranes, and 24 h for STRAT-M^®^, and then analysed by HPLC (Vanquish, Thermo-scientific, United States). The sampling volume was immediately replaced with the same volume of fresh PBS prewarmed solution at 37°C ± 0.5°C.

At the end, the concentration in the receiving chamber, the flux (g/s·cm^2^), and apparent permeability (cm/s) were determined using Equations 1, 2, as described in ref. 15.

$$J=\frac{dQ}{dt}A$$ (1)

$$P_{app}=\frac{J}{C_{0}}$$ (2)

where *J* is the flux through the membrane to the receptor compartment, *dQ* is the amount of drug across the membrane, *dt* is the permeation time (in seconds), and *A* is the diffusion area (in cm^2^), calculated from the radius of the Franz cell, which was 1.77 cm^2^. Note that J was obtained from the slope of the curve at steady state. The apparent permeability (P_app_) was calculated normalizing the flux (J) over the drug concentration in the donor compartment C_0_.

#### 2.2.4 Barrier effect studies

The barrier effect studies were performed with a Franz cell (Copley Scientific, United Kingdom), studying caffeine permeability through the membrane with respect to a negative and positive control. The untreated membrane was the negative control, while membrane covered with a Vaseline film was the positive one. The procedure was the same as the above-reported permeation studies with slight modifications: before filling the receiving chamber, in the donor, 200 mg of Vaseline (in the case of positive control) or 200 mg di PBS (in the case of negative control) was added over the membrane. In both cases, the added substances were left to equilibrate for 2 h before adding both the caffeine solution to the donor chamber and PBS to the receiving chamber (10.5 mL). Following this procedure, when applying the test to a real product, 200 mg of formulation can be spread over the membrane and the permeability data can then be compared with both the negative and positive ones. In all cases, the samples recovered from the receiving chamber were collected from 0 up to 3 h (0.5; 1.0; 1.5; 2.0; 3.0 h) and analysed by HPLC (Vanquish, Thermo-scientific, United States). The sampling volume was immediately replaced with the same volume of fresh PBS prewarmed solution at 37°C ± 0.5°C.

#### 2.2.5 Membrane integrity

The membrane integrity was assessed by colorimetric assay using methylene blue dye. This procedure was applied at the end of each test (i.e., permeability, positive controls, negative controls, and barrier effect tests with the studied product). After the test, the donor chamber was washed with 2 mL of PBS (2 times) and 1 mL of methylene blue solution 0.05% was added. After 1 h, the receiving chamber samples were qualitatively evaluated, to confirm the colorlessness of the receiving solution. For comparative purposes, the test was also performed on a damaged model-like membrane (polymeric support without phospholipidic bilayer functionalization) in which the dye permeates, forming a blue receiving solution.

### 2.3 Statistical analysis

All experiments were performed in at least tripled independent replicates. Values are reported as means with standard deviation (SD) of the average value. Statistical analysis was performed using Microsoft Excel.

## 3 Results and discussion

### 3.1 Analytical method validation

**FIGURE 1:**
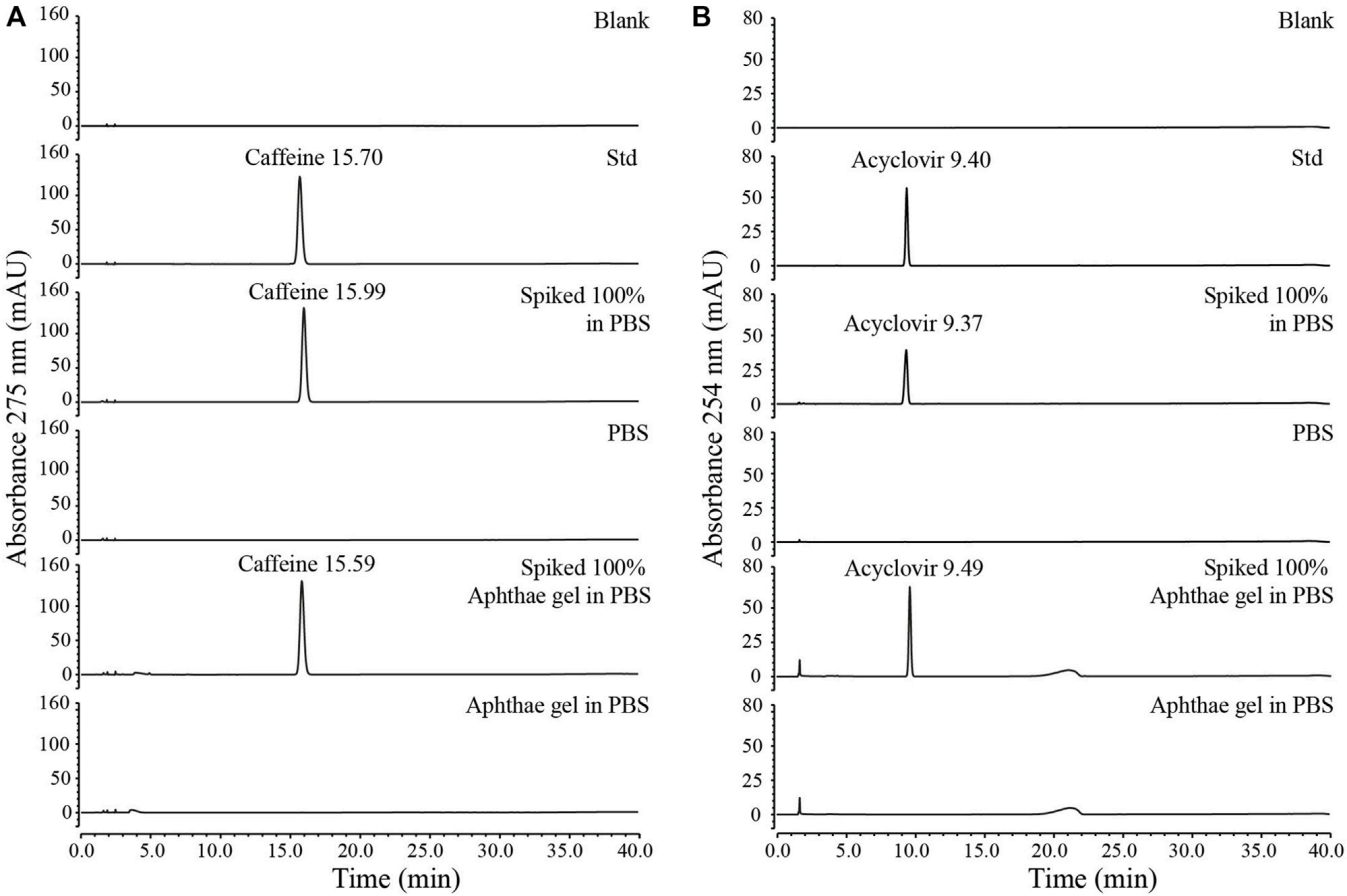
Specificity test for HPLC methods validation: **(A)** specificity test for caffeine HPLC method, **(B)** specificity test for acyclovir HPLC method.

The HPLC analytical methods for the quantification of caffeine and acyclovir were developed and validated by the determination of the following parameters: linearity, sensitivity, specificity, precision, and accuracy. The complete discussion, equations, figures, and dataset related to the analytical validation methods are reported in the Supplementary Material. In summary, the linear range for caffeine and acyclovir was 0.9–192 μg/mL and 0.1–50 μg/mL, respectively. Regarding the sensitivity, the limit of detection (LOD) of the analytical method was 0.028 μg/mL for caffeine and 0.01 μg/mL for acyclovir, while the limit of quantitation (LOQ) was 0.094 μg/mL for caffeine and 0.03 μg/mL for acyclovir. The methods specifically determined caffeine and acyclovir both in pure PBS solutions and in PBS with Aphthae gel. As a matter of fact, in both pure PBS and PBS with Aphthae gel, no interfering peaks that had the same retention time of caffeine and acyclovir were detected (Figure 1). Additionally, analyte chromatographic peak purity was confirmed by the analysis of the UV spectra recorded by DAD (data not shown). Lastly, precision and accuracy were evaluated by three replicate determinations of spiked samples at 80%, 100%, and 120% of the expected analyte concentration. The precision of the HPLC methods was determined as the percentage of relative standard deviation (RSD %, see equation (4S) in Supplementary Material) of the peak areas for replicate injections of the samples (*n* = 3 for each concentration). The mean RSD % for caffeine and acyclovir in pure PBS solutions were found to be 0.26% and 0.20%, respectively. On the other hand, the mean RSD % for caffeine and acyclovir with Aphthae gel in PBS solutions were found to be 0.09% and 0.11%, respectively. The obtained results indicated that the precision of analytical methods can be defined as acceptable, due to the RSD % of ≤2.0%. Additionally, the accuracy of the developed HPLC-UV methods was assessed *via* a recovery test. The mean Recovery % (see equation (5S) in Supplementary Material) of the analytical procedures for caffeine and acyclovir in PBS solutions were found to be 93.9% and 94.2%, respectively. On the other hand, the mean Recovery % of analytical procedures for caffeine and acyclovir with Aphthae gel in PBS solutions were found to be 97.6% and 109.7%, respectively. These results indicated that the accuracy can be defined as acceptable, owing to 80% ≤ % Recovery ≤120% for each concentration.

### 3.2 Experimental design and effect of biomimetic tissues

In accordance with Texeira and co-workers’ permeability test (Teixeira et al., 2020), caffeine and acyclovir were tested over the intestinal artificial biomimetic membrane to replicate the permeability values obtained and to extend the test also to different membranes. Caffeine was selected beacause it is considered a very highly permeable substance caffeine is considered a very highly permeable substance and it is a reference standard for barrier effect studies. Conversely, acyclovir was taken as the lowest reference standard, being a low permeable drug. The test was performed using a Franz cell as a vertical diffusion cell and an intestinal biomimetic artificial membrane, prepared in accordance with that previously described by Corti et al. (2006a). Data obtained were compared to those in the literature to confirm the correct implementation of the experimental protocol and to validate the developed method, the reproducibility, and the validity of the artificial biomimetic membranes as well.

**FIGURE 2:**
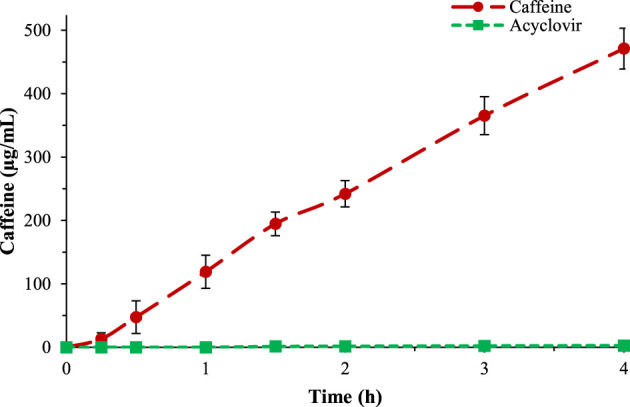
Comparison between caffeine (red) and acyclovir (green) permeability across the gastro-intestinal biomimetic membrane.

Caffeine and acyclovir permeability were evaluated through time and compared, resulting in a very high and very low apparent permeability value (30.5E-6 and 0.6E-6), respectively (Supplementary Table S15). The test was performed up to 6 h. The data obtained were plotted into a graph (Figure 2), in which it is possible to observe the differences between the behaviors of these two drugs across the gastro-intestinal membrane. In Supplementary Figures S3–S5, the HPLC spectra evolution over time, for caffeine and acyclovir, are shown respectively.

Another *in vitro* method to study the permeability of a compound consists of the use of cell-based methods. The ability of cells to create a barrier defines the ability of an assay to predict drug absorption. Several cell lines and culture systems were used to replicate a specific epithelium *in vivo* to predict drug absorption (Balimane et al., 2000). Monolayers have barrier properties (e.g., polarity, water interface, and tight junctions) under specific conditions that can be used for drug permeability experiments. Caco-2 are cells of human colon adenocarcinoma that exhibit many of the functional and morphological properties of the human intestinal enterocytes. They express a large part of the nutrient and drug transporter systems, as well as a portion of the metabolic enzymes expressed in the intestinal epithelium (Miret et al., 2004). The use of artificial biomimetic methods is a valid substitution to cell-based methods, and, in particular, several studies have compared the permeability of different drugs over both Caco-2 and intestinal biomimetic membrane, proving that, for those drugs that are transported just by passive diffusion (almost all drugs were absorbed by passive diffusion), the apparent permeability values can be comparable. To demonstrate this equivalence, the permeability data obtained in this study were also compared to Caco-2 permeability values (Yamashita et al., 2000; Zhu et al., 2002). In Table 1, the data obtained in this study were compared with Caco-2 and the Franz cell method literature data. The data obtained showed that the biomimetic membrane has a very similar permeability pattern in respect to cell-based tissue. Therefore, these simple and effective biomimetic membranes can be used as a valid alternative for studying the permeability performance of active substances.

**TABLE 1:** Comparison between experimental data and literature data.

	Franz cell (this study) cm/s	Franz cell (Teixeira et al., 2020) cm/s (E)	Caco-2 (Yamashita et al., 2000; Zhu et al., 2002) cm/s (E)
Caffeine	30.5E-6	36.2-6	30.8-6
Acyclovir	0.6E-06	0.40-6	0.3-6

Once the method was validated, the caffeine permeability was evaluated over different biomimetic artificial membranes.

**FIGURE 3:**
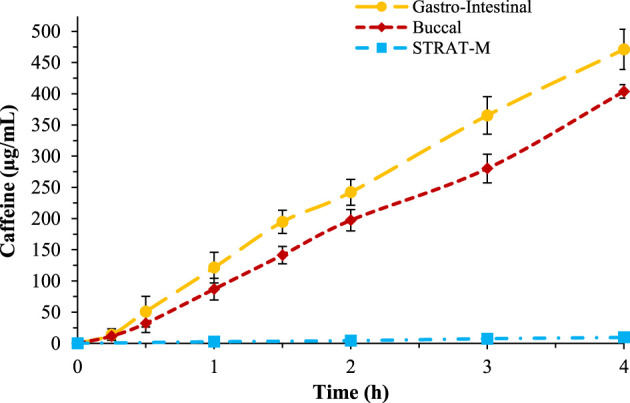
Comparison of caffeine permeability over gastro-intestinal membrane (yellow), buccal membrane (red), and STRAT-M^®^ (blue).

The buccal membranes were prepared following the indications of Mura et al. (2018). In detail, a lipidic ternary mixture composed of *n*-octanol, Lipoid^®^E80, and cholesterol was prepared and a cellulose acetate-nitrate membrane with pore size of 0.025 μm was impregnated and then used to perform the caffeine and acyclovir permeability tests. The tests were performed in the same conditions as the previous and the results showed that the buccal membrane is less permeable than the intestinal (Figure 3).

The same test was repeated also to study caffeine permeability across the skin, using the STRAT-M^®^ biomimetic membrane. The conditions used for the transdermal permeability were different to the previous tests; the temperature of the skin test was 32°C and the test was performed studying caffeine permeability up to 24 h (Figure 3). The results showed that caffeine has a very low permeability across this membrane, in accordance with literature data.

In Table 2, the P_app_ values are reported, in which it is possible to observe that the gastro-intestinal membrane is the most permeable and the transdermal the least. The data obtained are in accordance with *in vivo* tests. In Figure 3, the percentage of permeated caffeine in all three different membranes were reported as a comparison, showing a significative difference of caffeine permeation between the membranes (*p* < 0.05). In Supplementary Figure S6, the HPLC spectra for caffeine over the three different membranes were compared.

**TABLE 2:** Caffeine permeability in different artificial biomimetic membranes.

	Gastro-intestinal	Buccal	Dermal
P_app_ x10^−6^ cm/s	25.3	18.2	0.5

Taking advantage of the implementation of these three different artificial membranes to evaluate caffeine permeability, the barrier effect method was developed to study the performance of SB-MDs over different biomimetic compartments.

### 3.3 Barrier effect studies

#### 3.3.1 General protocol design

**FIGURE 4:**
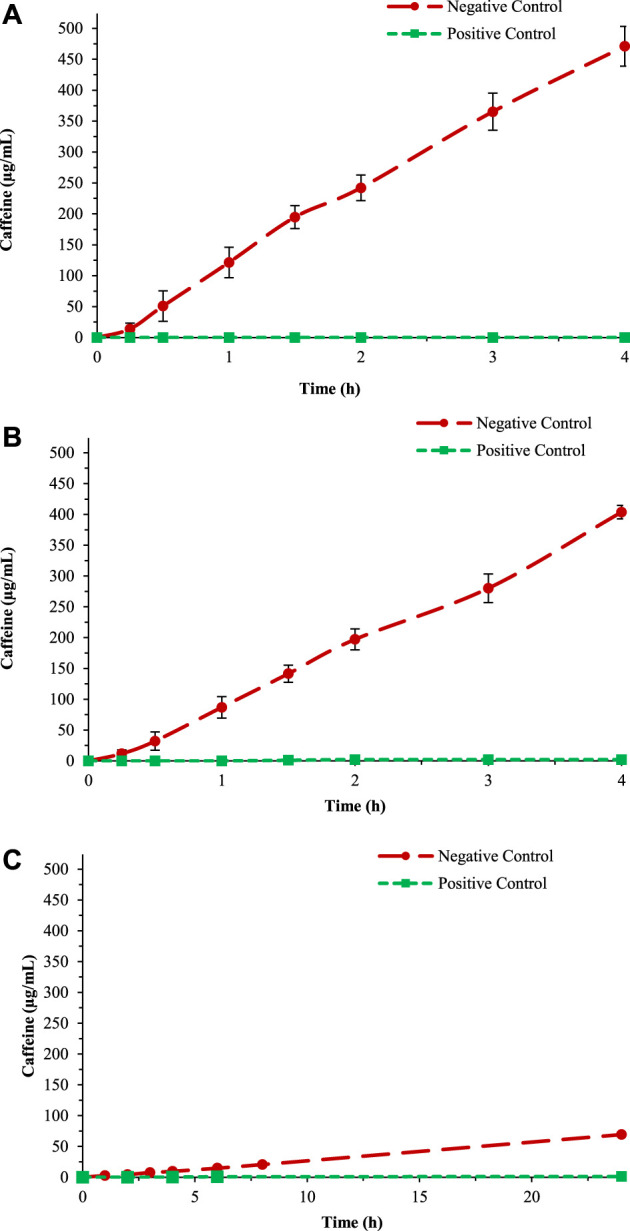
Negative and positive control test for the three different membranes: **(A)** Gastro-intestinal, **(B)** Buccal, and **(C)** STRAT-M^®^.

The barrier effect is an *in vitro* test useful to determine the performance of a medical device and permit identification of their film-forming ability. For the *in vitro* test, caffeine was selected as a probe to assess the propensity of a given product to form a protective film due to its ability to permeate different models of human tissues (i.e., intestinal, buccal, and cutaneous) even in absence of damage. The barrier effect test consists of studying the caffeine permeability over an artificial biomimetic membrane covered by the SB-MD and comparing the permeability with a positive and negative control. For the positive control, the biomimetic membrane was covered with Vaseline, a substance able to create a strong protective film, through which caffeine is not able to pass. For the negative control, the permeation of caffeine was evaluated after treating the membrane with PBS solution (Figure 4). The complete positive and negative control dataset together with chromatograms are reported in Supplementary Tables S16–S18 and Supplementary Figures S7–S9.

For the formation of the protective film over the membrane, the substance was left for 2 h before the barrier effect test started. All tests were done five times to evaluate the reproducibility and accuracy of data. At the end of each test, the integrity of the membrane was evaluated: the presence of colourless receiving solution indicated the absence of damage in the membrane structure and, therefore, a significative permeability data.

The method as developed will permit the evaluation of the barrier effect of SB-MDs. With this aim, caffeine permeability across the “untreated” membrane may be normalized as 100%, and the difference in terms of caffeine permeability in respect to both negative and positive controls (as described above) will numerically measure the film-forming ability of the formulation based on the substances under examination. The pattern of the protocol is shown in Supplementary Figure S10.

#### 3.3.2 Case study: Barrier effect of aphthae gel

Aphthae gel is an experimental gel-like SB-MD under study for the treatment of aphthae, stomatitis, and microlesions of the mouth and which, besides the excipients, contains xyloglucan, aloe vera extract, vegetal natural glycerol, and polyvinylpyrrolidone (PVP K30) as active ingredients. All these functional components have hydrating, protective, lenitive, and adhesive properties, as well as film-forming ability (Nair, 1998; Sharma et al., 2014; Esquena-Moret, 2022). Herein, the barrier effect of Aphthae gel was determined by applying the protocol developed. To test the product, the absorption of caffeine was evaluated across a synthetic biomimetic buccal membrane treated with 200 mg of Aphthae gel. The test was done in triplicate to evaluate the reproducibility and accuracy of data. In Table 3, the average data with SD are reported.

**TABLE 3:** Aphthae gel—Concentration data of the caffeine passage.

Time	Average data with SD
[h]	[µg/mL] ± *Δ*_Conc_
0	0.000 ± 0.000
0.5	5.497 ± 0.572
1	22.270 ± 2.954
1.5	40.119 ± 3.488
2	66.443 ± 2.546
3	109.700 ± 3.782
**Membrane integrity test**	**Compliant**

**FIGURE 5:**
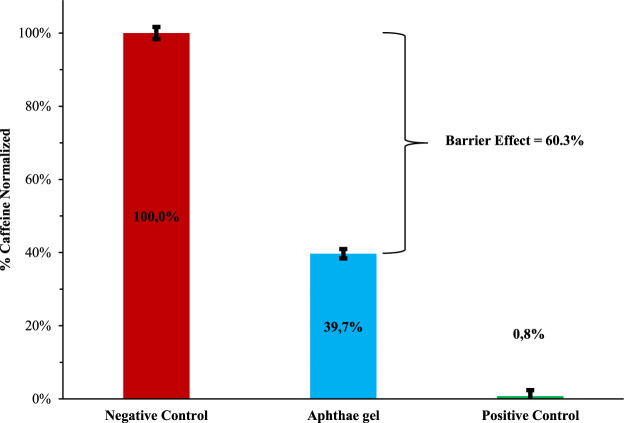
Barrier effect of Aphthae gel—comparison between value at 3 h of experiment.

To calculate the reduction of caffeine passage, the values at 3 h were plotted into a histogram in comparison with the positive and negative control (as above described). The percentage of caffeine in the negative control obtained using the buccal membrane (data reported in Figure 4B) has been considered 100% (Figure 5).

After treatment with Aphthae gel, a reduction of 60.3% in caffeine permeability across a biomimetic membrane was observed.

## 4 Conclusion

This study had the goal of developing a standard barrier effect procedure useful to evaluate the film-forming ability of SB-MDs as a part of the experimental process necessary to establish the safety and effectiveness of the products in response to the new European regulations and in accordance with animal welfare aspects (principle of the 3Rs).

With this aim, in a first validating approach, the permeability of model drugs (caffeine and acyclovir) was studied over biomimetic membranes (simulating gastro-intestinal, buccal, and skin tissues). The results obtained were compared with literature data confirming a) the correct implementation of the experimental protocol, and b) the procedure of the artificial biomimetic membrane. The results of the study of the lipid-impregnated membranes replicated the data obtained from the literature, showing a significative difference in terms of caffeine permeability between the three different biomimetic membranes. The gastro-intestinal support showed an apparent permeability higher than both buccal and skin, as expected. In the second part of the study, we demonstrated that these simple and effective biomimetic membranes may be successfully exploited to develop a barrier effect *in vitro* test to evaluate the protecting performance of substance-based medical devices (SB-MD). The protocols here proposed were designed by comparing the ability of a SB-MD to reduce the permeation of caffeine through a tissue (pre-covered with the product under examination), with positive (untreated tissue) and negative (tissue covered with Vaseline) controls. The barrier effect was expressed as the difference in terms of caffeine permeability between negative controls (normalized as 100%) and the SB-MD. The designed *in vitro* test was then applied to Aphthae gel, an experimental gel-like SB-MD under development and herein used as a case study. The results showed that the product reduces caffeine permeability across a biomimetic buccal membrane by about 60.3%. This data highlights the capability of Aphthae gel to protect the mucosae. Overall, the results of this study show that biomimetic membranes represent a useful tool for the preliminary high throughput screening of film-forming formulation candidates to be further tested for their efficacy and safety in clinical trials, as requested by the regulations related to substance-based medical devices (SB-MD). Concluding, this work provides scientifically validated procedures which may contribute to the creation of standard methods to assess the biological evaluation of medical devices.

## Data Availability

The original contribution presented in the study are included in the article/Supplementary Material, further inquiries can be directed to the corresponding author.

## References

[B1] Balimane P. Chong S. Morrison R. A. (2000). Current methodologies used for evaluation of intestinal permeability and absorption. J. Pharmacol. Toxicol. Methods 44 (1), 301–312. PMID: 11274897. 10.1016/s1056-8719(00)00113-1 11274897

[B2] Berben P. Bauer-Brandl A. Brandl M. Faller B. Flaten G. E. Jacobsen A. C. (2018). Drug permeability profiling using cell-free permeation tools: Overview and applications. Eur. J. Pharm. Sci. 119, 219–233. Elsevier B.V. 10.1016/j.ejps.2018.04.016 29660464

[B3] Casiraghi A. Ranzini F. Musazzi U. M. Franzè S. Meloni M. Minghetti M. (2017). *In vitro* method to evaluate the barrier properties of medical devices for cutaneous use. Regul. Toxicol. Pharmacol. 90, 42–50. 10.1016/j.yrtph.2017.08.007 28822878

[B4] Corti G. Maestrelli F. Cirri M. Furlanetto S. Mura P. (2006). Development and evaluation of an *in vitro* method for prediction of human drug absorption: I. Assessment of artificial membrane composition. Eur. J. Pharm. Sci. 27 (4), 346–353. 10.1016/j.ejps.2005.11.004 16359848

[B5] Corti G. Maestrelli F. Cirri M. Zerrouk N. Mura P. (2006). Development and evaluation of an *in vitro* method for prediction of human drug absorption: II. Demonstration of the method suitability. Eur. J. Pharm. Sci. 27 (4), 354–362. 10.1016/j.ejps.2005.11.005 16364612

[B6] di Cagno M. Bibi H. A. Bauer-Brandl A. (2015). New biomimetic barrier Permeapad^TM^ for efficient investigation of passive permeability of drugs. Eur. J. Pharm. Sci. 73, 29–34. 10.1016/j.ejps.2015.03.019 25840123

[B7] Di L. Artursson P. Avdeef A. Ecker G. F. Faller B. Fischer H. (2012). Evidence-based approach to assess passive diffusion and carrier-mediated drug transport. Drug Discov. 17 (15–16), 905–912. 10.1016/j.drudis.2012.03.015 22507594

[B8] Eeman M. Deleu M. (2010). From biological membranes to biomimetic model membranes. Biotechnol. *Agron. Société Environ*. 14 (4), 719–736.

[B9] Esquena-Moret J. (2022). A review of xyloglucan: Self-aggregation, hydrogel formation, mucoadhesion and uses in medical devices. Macromol 2, 562–590. 10.3390/macromol2040037

[B10] Fedi A. Vitale C. Ponschin G. Ayehunie S. Fato M. Scaglione S. (2021). *In vitro* models replicating the human intestinal epithelium for absorption and metabolism studies: A systematic review. Control. Release 335, 247–268. 10.1016/j.jconrel.2021.05.028 34033859

[B11] Fimognari C. Barrajón-Catalán E. Luceri C. Turrini E. Raschi E. Bigagli E. (2022). New regulation on medical devices made of substances: Opportunities and challenges for pharmacological and toxicological research. Front. Drug Saf. Regul. 2, 1001614. 10.3389/fdsfr.2022.1001614

[B12] Giovagnoni E. (2022). Substance-based medical devices made of natural substances: An opportunity for therapeutic innovation. Front. Drug Saf. Regul. 2, 998114. 10.3389/fdsfr.2022.998114

[B13] Haq A. Dorrani M. Goodyear B. Joshi V. Michniak-Kohn B. (2018). Membrane properties for permeability testing: Skin versus synthetic membranes. Int. J. Pharm. 539 (1–2), 58–64. 10.1016/j.ijpharm.2018.01.029 29366943

[B14] Haq A. Goodyear B. Ameen D. Joshi V. Michniak-Kohn B. (2018). Strat-M® synthetic membrane: Permeability comparison to human cadaver skin. Int. J. Pharm. 547 (1–2), 432–437. 10.1016/j.ijpharm.2018.06.012 29890259

[B15] Hopf N. B. Champmartin C. Schenk L. Berthet A. Chedik L. Du Plessis J. L. (2020). Reflections on the OECD guidelines for *in vitro* skin absorption studies. Regul. Toxicol. Pharmacol. 117, 104752. 10.1016/j.yrtph.2020.104752 32791089

[B16] ISO (2020). Biological evaluation of medical devices. Available at: ISO - 11.100.20 - Biological evaluation of medical devices.

[B17] Khdair A. Hamad I. Al-Hussaini M. Albayati D. Alkhatib H. Alkhalidi B. (2013). *In vitro* artificial membrane-natural mucosa correlation of carvedilol buccal delivery. J. *Drug Deliv. Sci. Technol*. 23 (6), 603–609. 10.1016/s1773-2247(13)50092-x

[B18] Loftsson T. Konrádsdóttir F. Másson M. (2006). Development and evaluation of an artificial membrane for determination of drug availability. Int. J. Pharm. 326 (1–2), 60–68. 10.1016/j.ijpharm.2006.07.009 16920289

[B19] Manellari S. Leone M. Casiraghi A. Gennari C. Minghetti P. (2022). Medicinal products, medical devices, or accessories of medical devices: How to qualify gases for spirometry? Front. Drug Saf. Regul. 2, 1089965. 10.3389/fdsfr.2022.1089965

[B20] Miret S. Abrahamse L. de Groene E. M. (2004). Comparison of *in vitro* models for the prediction of compound absorption across the human intestinal mucosa. J. Biomol. Screen. 9 (7), 598–606. 10.1177/1087057104267162 15475479

[B21] Mura P. Orlandini S. Cirri M. Maestrelli F. Mennini N. Casella G. (2018). A preliminary study for the development and optimization by experimental design of an *in vitro* method for prediction of drug buccal absorption. Int. J. Pharm. 547 (1–2), 530–536. 10.1016/j.ijpharm.2018.06.032 29908330

[B22] Nair B. (1998). Final report on the safety assessment of polyvinylpyrrolidone (PVP). Int. J. Toxicol. 17 (4), 95–130. 10.1177/109158189801700408

[B23] Ng S. F. Rouse J. J. Sanderson F. D. Meidan V. Eccleston G. M. (2010). Validation of a static Franz diffusion cell system for *in vitro* permeation studies. AAPS PharmSciTech 11 (3), 1432–1441. 10.1208/s12249-010-9522-9 20842539 PMC2974154

[B24] OECD (2004). “Test No. 428: Skin absorption,” *in Vitro* method, *OECD Guidelines for the Testing of chemicals*, section 4 (Paris: OECD Publishing). 10.1787/9789264071087-en

[B25] Pellegatta G. Spadaccini M. Lamonaca L. Craviotto V. D’amico F. Ceriotti L. (2020). Evaluation of human esophageal epithelium permeability in presence of different formulations containing hyaluronic acid and chondroitin sulphate. Med. Devices Evid. Res. 13, 57–66. 10.2147/MDER.S234810 PMC7069498 32210642

[B26] Salamanca C. H. Barrera-Ocampo A. Lasso J. C. Camacho N. Yarce C. J. (2018). Franz diffusion cell approach for pre-formulation characterisation of ketoprofen semi-solid dosage forms. Pharmaceutics 10 (3), 148–210. 10.3390/pharmaceutics10030148 30189634 PMC6161298

[B27] SCCP (2010). Opinion on basic citeria for the *in vitro* assessment of dermal absorption of cosmetic ingredients. Scientific Committee on Consumer Safety. Sccp/0970/06 2006, No. March, 27–38.

[B28] Sharma P. Kharkwal A. London H. K. Abdin M. Varma A. (2014). A review on pharmacological properties of aloe vera. Int. J. Pharm. Sci. Rev. Res. 29, 31–37.

[B29] Teixeira L. S. Chagas T. V. Alonso A. Gonzalez‐alvarez I. Bermejo M. Polli J. (2020). Biomimetic artificial membrane permeability assay over franz cell apparatus using bcs model drugs. Pharmaceutics 12 (10), 988–1016. 10.3390/pharmaceutics12100988 33086670 PMC7589491

[B30] UE (2021). UE 2017/745 entered into force. 26 May 2021.

[B31] Yamashita S. Furubayashi T. Kataoka M. Sakane T. Sezaki H. Tokuda H. (2000). Optimized conditions for prediction of intestinal drug permeability using Caco-2 cells. Eur. J. Pharm. Sci. 10, 195–204. 10.1016/s0928-0987(00)00076-2 10767597

[B32] Zhu C. Jiang L. Chen T. M. Hwang K. K. (2002). A comparative study of artificial membrane permeability assay for high throughput profiling of drug absorption potential. Eur. J. Med. Chem. 37, 399–407. 10.1016/s0223-5234(02)01360-0 12008054

