# Supplementary material for: Safety and efficacy of substance-based medical devices: Design of an in vitro barrier effect test
Source: Front Drug Saf Regul. 2023 Apr 11;3:1124873. doi: 10.3389/fdsfr.2023.1124873 (PMC12443116; doi:10.3389/fdsfr.2023.1124873)
Supplement: Supplementary file 1 [file DataSheet1.docx]

Supplementary Material

# Supplementary Data

## Analytical Method validation

### Linearity

The Caffeine and Acyclovir calibration curves were obtained using some standard solutions in different range concentrations of Caffeine (from 0.9 to 192 µg/mL) and Acyclovir (from 0.1 to 50 µg/mL). Three independent injections of each sample were carried out. Peak area values of the standard were plotted against the concentrations. The methods linearity was valuated as a correlation coefficient by linear regression analysis.

The linear equation for Caffeine was:

$\boldsymbol{y = 0.4878 x - 0.0842;}\boldsymbol{R}^{\boldsymbol{2}}\boldsymbol{= 0.9999}$ **(**Equation 1S)

and for Acyclovir was:

$\boldsymbol{y = 1.0932 x -0.0852;}\boldsymbol{R}^{\boldsymbol{2}}\boldsymbol{=1.0000}$ **(**Equation 2S)

The calibration curves are shown in Figure 1S and Figure 2S and data are reported in Table 1S and Table 2S. The linear range for Caffeine and Acyclovir was (0.9-192 μg/mL) and (0.1-50 µg/mL) respectively.

Table 1S. Results of calibration curve analysis for Caffeine HPLC method.

| **Sample ID** | **Concentration** | **Area** | | | **Mean Area** | **Mean Ret. Time** |
| --- | --- | --- | --- | --- | --- | --- |
| **Caffeine** | **(µg/mL)** | **(mAU*min)** | | | **(mAU*min)** | **(min)** |
| Std1 - 1% | 0.94 | 0.468 | 0.467 | 0.460 | 0.465 | 15.859 |
| Std2 - 5% | 4.68 | 2.231 | 2.216 | 2.226 | 2.224 | 15.835 |
| Std3 - 10% | 9.63 | 4.773 | 4.77 | 4.766 | 4.770 | 15.820 |
| Std4 - 25% | 24.07 | 11.883 | 11.878 | 11.879 | 11.880 | 15.805 |
| Std5 - 50% | 48.16 | 22.619 | 22.613 | 22.604 | 22.612 | 15.797 |
| Std6 - 80% | 77.06 | 37.683 | 37.672 | 37.682 | 37.679 | 15.786 |
| Std7 - 100% | 93.50 | 45.328 | 45.372 | 45.319 | 45.340 | 15.769 |
| Std8 - 120% | 115.60 | 56.607 | 56.627 | 56.602 | 56.612 | 15.755 |
| Std9 - 200% | 192.60 | 93.883 | 93.868 | 93.831 | 93.861 | 15.717 |

Figure 1S. Caffeine calibration curve

Table 2S. Results of calibration curve analysis for Acyclovir HPLC method.

| **Sample ID** | **Concentration** | **Area** | | | **Mean Area** | **Mean Ret. Time** |
| --- | --- | --- | --- | --- | --- | --- |
| **Acyclovir** | **(µg/mL)** | **(mAU*min)** | | | **(mAU*min)** | **(min)** |
| Std1 - 1% | 0.10 | 0.121 | 0.115 | 0.117 | 0.118 | 9.498 |
| Std2 - 5% | 0.50 | 0.541 | 0.544 | 0.540 | 0.542 | 9.381 |
| Std3 - 10% | 1.00 | 1.071 | 1.064 | 1.066 | 1.067 | 9.392 |
| Std4 - 50% | 5.00 | 5.287 | 5.289 | 5.284 | 5.287 | 9.383 |
| Std5 - 100% | 10.00 | 10.702 | 10.699 | 10.690 | 10.697 | 9.405 |
| Std6 - 250% | 25.00 | 27.196 | 27.196 | 27.189 | 27.194 | 9.400 |
| Std7 - 500% | 50.00 | 54.667 | 54.633 | 54.618 | 54.639 | 9.398 |

Figure 2S. Acyclovir calibration curve

### Sensitivity

The sensitivity of the methods was evaluated by determining the detection limit (LOD) and the quantitation (LOQ). The LOD is defined as the lowest concentration of an analyte in a sample that can be detected, but not necessarily quantified. On the contrary, LOQ is the lowest concentration of an analyte in a sample that can be determined with acceptable precision and accuracy. [^[[1]](#endnote-1)^] In this study LOD and LOQ were determined by using signal-to-noise method specified in the European Pharmacopoeia.[^[[2]](#endnote-2)^] The signal-to-noise (S/N) ratio was calculated from Equation (3S).

$\frac{\boldsymbol{S}}{\boldsymbol{N}}\boldsymbol{=}\frac{\boldsymbol{2}\boldsymbol{H}}{\boldsymbol{h}}$ **(**Equation 3S)

where *H* is the analyte peak’s height and *h* is the maximum height of blank signal magnitude. For the LOD was evaluated a S/N ratio of at least 3, while LOQ was established for an analyte concentration with a S/N ratio of at least 10.

The LOD of the analytical method was 0.028 µg/mL for Caffeine and 0.01 µg/mL for Acyclovir. Besides, LOQ was 0.094 µg/mL for Caffeine and 0.03 µg/mL for Acyclovir. Complete dataset for S/N values are reported in Table 3S and Table 4S.

Table 3S. Sensitivity results analysis for determination of LOD and LOQ of Caffeine HPLC method.

| **Sample ID** | | **Concentration** | **Height** | | | **Mean Height** | **S/N** | **Limit** |
| --- | --- | --- | --- | --- | --- | --- | --- | --- |
| **Caffeine** | **(µg/mL)** | | **(mAU)** | | | **(mAU)** |  |  |
| Blank | **-** | | 0.057 | 0.026 | 0.024 | 0.036 | - | - |
| Std1 - 1% | 1 | | 1.377 | 1.382 | 1.373 | 1.377 | 76 | - |
| Std0.1 – 0.1% | 0.094 | | 0.193 | 0.198 | 0.195 | 0.195 | 11 | LOQ |
| Std0.05 – 0.05% | 0.047 | | 0.133 | 0.113 | 0.129 | 0.125 | 7 | - |
| Std0.03 – 0.03% | 0.028 | | 0.103 | 0.101 | 0.105 | 0.103 | 5.8 | LOD |

Table 4S. Sensitivity results analysis for determination of LOD and LOQ of Acyclovir HPLC method.

| **Sample ID** | **Concentration** | **Height** | | | **Mean Height** | **S/N** | **Limit** |
| --- | --- | --- | --- | --- | --- | --- | --- |
| **Acyclovir** | **(µg/mL)** | **(mAU)** | | | **(mAU)** |  |  |
| Blank | **-** | 0.030 | 0.030 | 0.020 | 0.027 | - | - |
| Std1 - 1% | 0.1 | 0.590 | 0.590 | 0.580 | 0.587 | 43 | - |
| Std0.1 – 0.5% | 0.05 | 0.290 | 0.270 | 0.290 | 0.283 | 21 | - |
| Std0.05 – 0.3% | 0.03 | 0.160 | 0.160 | 0.160 | 0.160 | 12 | LOQ |
| Std0.03 – 0.1% | 0.01 | 0.060 | 0.060 | 0.070 | 0.063 | 4.8 | LOD |

### Specificity

Specificity of the developed HPLC-UV methods was determined in order to assess the matrix effect. To evaluate the specificity, analyte free formulation samples and analyte spike added to the formulation samples were carried out through the analytical procedure. The retention times of the matrix were compared with Caffeine and Acyclovir retention times.

### Precision and accuracy

Precision and accuracy were evaluated by three replicate determinations of spike samples at 80%, 100% and 120% of expected analyte concentration.

The precision of the HPLC methods was determined as percentage relative standard deviation (% RSD) of the peak areas for replicate injections of the samples (*n* = 3 for each concentration). The repeatability results and the relative standard deviation are reported in Table 5S, Table 6S, Table 7S, Table 8S. The RSD % was calculated from Equation (6S).

$\boldsymbol{RSD}\boldsymbol{\%=}\frac{\boldsymbol{SD}}{\boldsymbol{Mean} \boldsymbol{Value}} \boldsymbol{100}$ **(**Equation 4S)

where *SD* is the standard deviation and *Mean* is the mean value of the derived concentration from triplicate injection.

The mean RSD % for Caffeine and Acyclovir in pure PBS solutions were found to be 0.26% and 0.20 %, respectively. On the other hand, the mean RSD % for Caffeine and Acyclovir with Aphthae gel in PBS solutions were found to be 0.09% and 0.11 %, respectively. The obtained results indicate that precision of analytical methods can be defined acceptable, due to RSD % is ≤ 2.0%.

Table 5S. Results of precision analysis for Caffeine HPLC method.

| **Sample ID** | **Concentration** | **Quantity** | | | **Mean Quantity** | **SD** | **RSD** |
| --- | --- | --- | --- | --- | --- | --- | --- |
| **Caffeine** | **(µg/mL)** | **(µg/mL)** | | | **(µg/mL)** |  | **(%)** |
| PBS - Spike 80% | 80 | 75.289 | 74.973 | 74.610 | 74.957 | 0.340 | 0.453 |
| PBS - Spike 100% | 100 | 95.019 | 94.982 | 94.935 | 94.979 | 0.042 | 0.044 |
| PBS - Spike 120% | 120 | 112.079 | 111.557 | 111.549 | 111.728 | 0.304 | 0.272 |

Table 6S. Results of precision analysis for Acyclovir HPLC method.

| **Sample ID** | **Concentration** | **Quantity** | | | **Mean Quantity** | **SD** | **RSD** |
| --- | --- | --- | --- | --- | --- | --- | --- |
| **Acyclovir** | **(µg/mL)** | **(µg/mL)** | | | **(µg/mL)** |  | **(%)** |
| PBS - Spike 80% | 8 | 7.297 | 7.262 | 7.239 | 7.266 | 0.029 | 0.402 |
| PBS - Spike 100% | 10 | 9.194 | 9.184 | 9.181 | 9.186 | 0.007 | 0.074 |
| PBS - Spike 120% | 12 | 11.989 | 12.019 | 12.014 | 12.007 | 0.016 | 0.134 |

Table 7S. Results of precision analysis for Caffeine HPLC method of Aphthae gel case study.

| **Sample ID** | **Concentration** | **Quantity** | | | **Mean Quantity** | **SD** | **RSD** |
| --- | --- | --- | --- | --- | --- | --- | --- |
| **Caffeine** | **(µg/mL)** | **(µg/mL)** | | | **(µg/mL)** |  | **(%)** |
| Aphthae gel - Spike 80% | 80 | 78.105 | 78.081 | 77.794 | 77.994 | 0.173 | 0.222 |
| Aphthae gel - Spike 100% | 100 | 97.472 | 97.469 | 97.458 | 97.466 | 0.007 | 0.007 |
| Aphthae gel - Spike 120% | 120 | 117.615 | 117.563 | 117.526 | 117.568 | 0.045 | 0.038 |

Table 8S. Results of precision analysis for Acyclovir HPLC method of Aphthae gel case study.

| **Sample ID** | **Concentration** | **Quantity** | | | **Mean Quantity** | **SD** | **RSD** |
| --- | --- | --- | --- | --- | --- | --- | --- |
| **Acyclovir** | **(µg/mL)** | **(µg/mL)** | | | **(µg/mL)** |  | **(%)** |
| Aphthae gel - Spike 80% | 8 | 8.602 | 8.579 | 8.568 | 8.583 | 0.017 | 0.202 |
| Aphthae gel - Spike 100% | 10 | 10.847 | 10.850 | 10.856 | 10.851 | 0.004 | 0.041 |
| Aphthae gel - Spike 120% | 12 | 13.596 | 13.603 | 13.616 | 13.605 | 0.010 | 0.076 |

Besides, accuracy of the developed HPLC-UV methods was assessed via a recovery test.

The recovery of added standard to PBS solutions was determined in triplicate analysis and calculated from Equation (5S):

$\boldsymbol{Recovery \%=}\frac{\boldsymbol{V}_{\boldsymbol{exp}}}{\boldsymbol{V}_{\boldsymbol{real}}}\boldsymbol{100}$ **(**Equation 5S)

where *V_exp_* is the experimental value and *V_real_* is the expected value of the derived concentration from triplicate analysis. Accuracy results were reported in Table 9S, Table 10S, Table 11S, Table 12S.

Table 9S. Results of accuracy analysis for Caffeine HPLC method.

| **Sample ID** | **Concentration** | **Quantity** | | | **SD** | **Mean Quantity** | **Recovery** | **Mean Recovery** |
| --- | --- | --- | --- | --- | --- | --- | --- | --- |
| **Caffeine** | **(µg/mL)** | **(µg/mL)** | | |  | **(µg/mL)** | **(%)** | **(%)** |
| PBS - Spike 80% | 80 | 75.289 | 74.973 | 74.610 | 0.340 | 74.957 | 93.7 | **93.9** |
| PBS - Spike 100% | 100 | 95.019 | 94.982 | 94.935 | 0.042 | 94.979 | 95.0 |  |
| PBS - Spike 120% | 120 | 112.079 | 111.557 | 111.549 | 0.304 | 111.728 | 93.1 |  |

Table 10S. Results of accuracy analysis for Acyclovir HPLC method.

| **Sample ID** | **Concentration** | **Quantity** | | | **SD** | **Mean Quantity** | **Recovery** | **Mean Recovery** |
| --- | --- | --- | --- | --- | --- | --- | --- | --- |
| **Acyclovir** | **(µg/mL)** | **(µg/mL)** | | |  | **(µg/mL)** | **(%)** | **(%)** |
| PBS - Spike 80% | 8 | 7.297 | 7.262 | 7.239 | 0.029 | 7.266 | 90.8 | **94.2** |
| PBS - Spike 100% | 10 | 9.194 | 9.184 | 9.181 | 0.007 | 9.186 | 91.9 |  |
| PBS - Spike 120% | 12 | 11.989 | 12.019 | 12.014 | 0.016 | 12.007 | 100.1 |  |

Table 11S. Results of accuracy analysis for Caffeine HPLC method of Aphthae gel case study.

| **Sample ID** | **Concentration** | **Quantity** | | | **SD** | **Mean Quantity** | **Recovery** | **Mean Recovery** |
| --- | --- | --- | --- | --- | --- | --- | --- | --- |
| **Caffeine** | **(µg/mL)** | **(µg/mL)** | | |  | **(µg/mL)** | **(%)** | **(%)** |
| Aphthae gel - Spike 80% | 80 | 78.015 | 78.081 | 77.794 | 0.173 | 77.994 | 97.5 | **97.6** |
| Aphthae gel - Spike 100% | 100 | 97.472 | 97.469 | 97.458 | 0.007 | 97.466 | 97.5 |  |
| Aphthae gel - Spike 120% | 120 | 117.615 | 117.563 | 117.526 | 0.045 | 117.568 | 98.0 |  |

Table 12S. Results of accuracy analysis for Acyclovir HPLC method of Aphthae gel case study.

| **Sample ID** | **Concentration** | **Quantity** | | | **SD** | **Mean Quantity** | **Recovery** | **Mean Recovery** |
| --- | --- | --- | --- | --- | --- | --- | --- | --- |
| **Acyclovir** | **(µg/mL)** | **(µg/mL)** | | |  | **(µg/mL)** | **(%)** | **(%)** |
| Aphthae gel - Spike 80% | 8 | 8.602 | 8.579 | 8.568 | 0.017 | 8.583 | 107.3 | **109.7** |
| Aphthae gel - Spike 100% | 10 | 10.847 | 10.850 | 10.856 | 0.004 | 10.851 | 108.5 |  |
| Aphthae gel - Spike 120% | 12 | 13.596 | 13.603 | 13.616 | 0.010 | 13.605 | 113.4 |  |

The mean Recovery % of analytical procedures for Caffeine and Acyclovir in PBS solutions were found to be 93.9 % and 94.2 %, respectively. On the other side, the mean Recovery % of analytical procedures for Caffeine and Acyclovir with Aphthae gel in PBS solutions were found to be 97.6 % and 109.7 %, respectively. The results indicate that accuracy can be defined acceptable owing to 80% ≤ % Recovery ≤ 120% for each concentration.

# Supplementary Figures and Tables

## Supplementary Figures


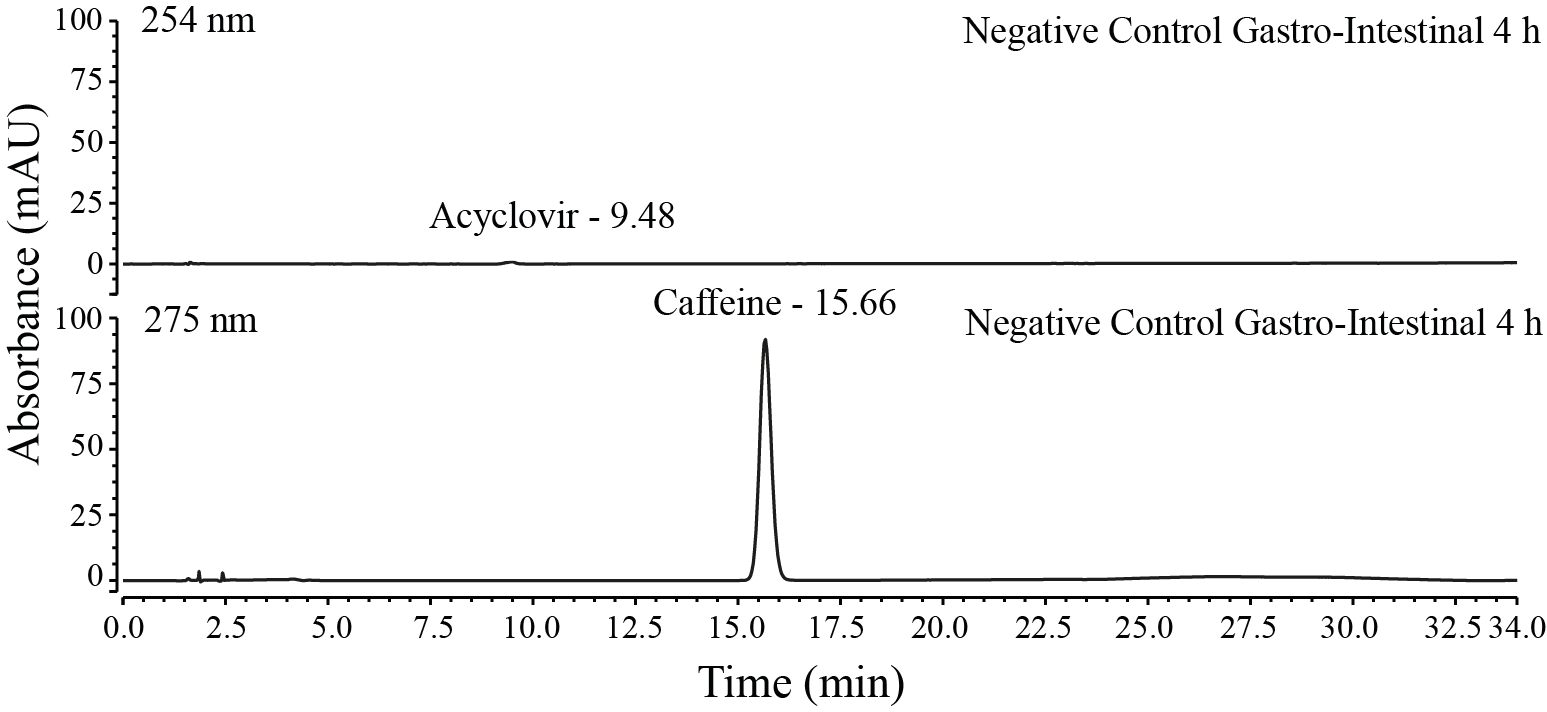


Figure 3S. Comparison of negative controls of gastro-intestinal membrane using Acyclovir (above) and Caffeine.


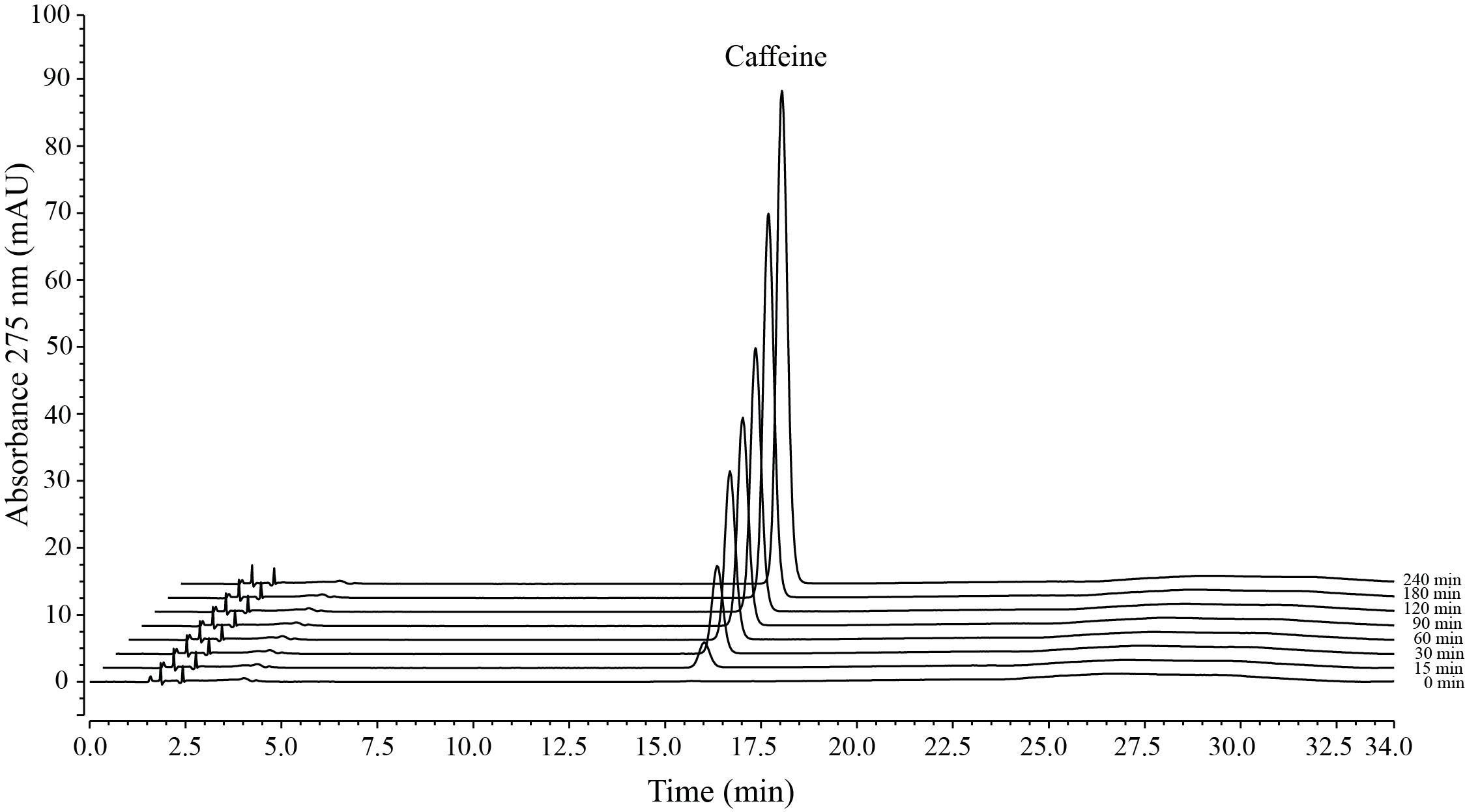


Figure 4S. HPLC analysis superposition from 0 to 240 min for Caffeine with negative gastro-intestinal membrane.


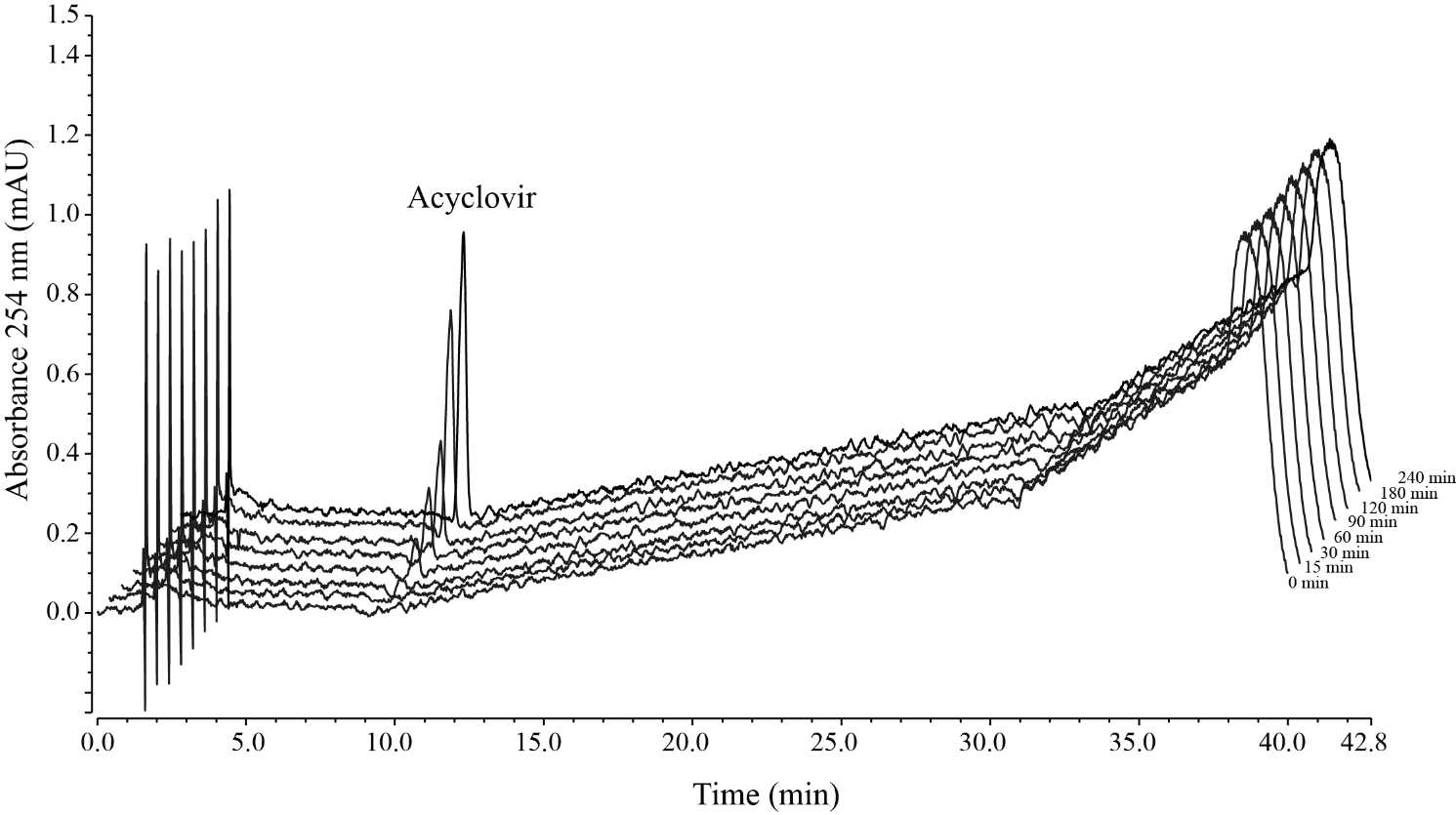


Figure 5S. HPLC analysis superposition from 0 to 240 min for Acyclovir with negative gastro-intestinal membrane.


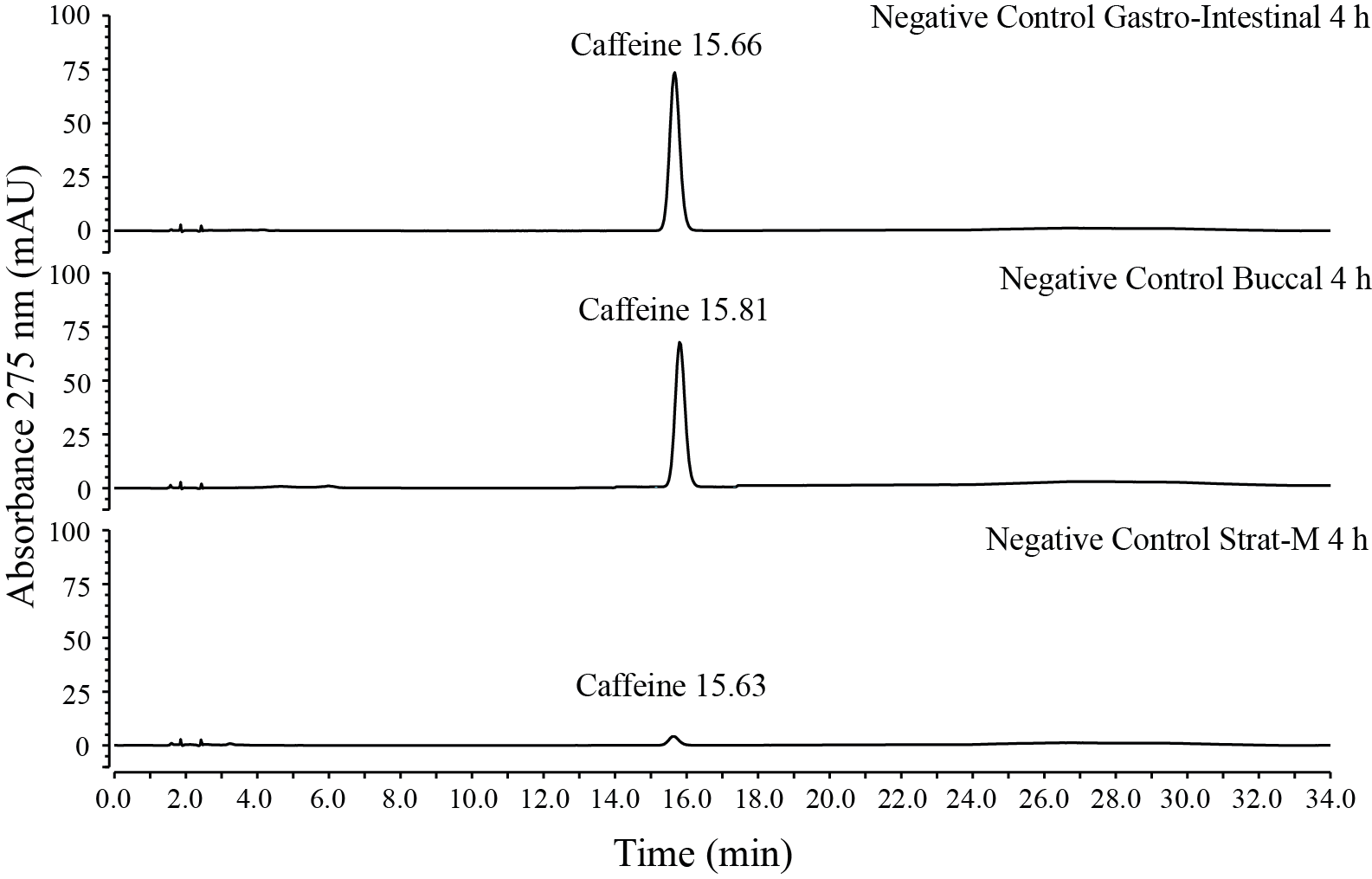


Figure 6S. Comparison of negative controls at 4 hours using: gastro-intestinal membranes, buccal membrane and STRAT-M^®^.


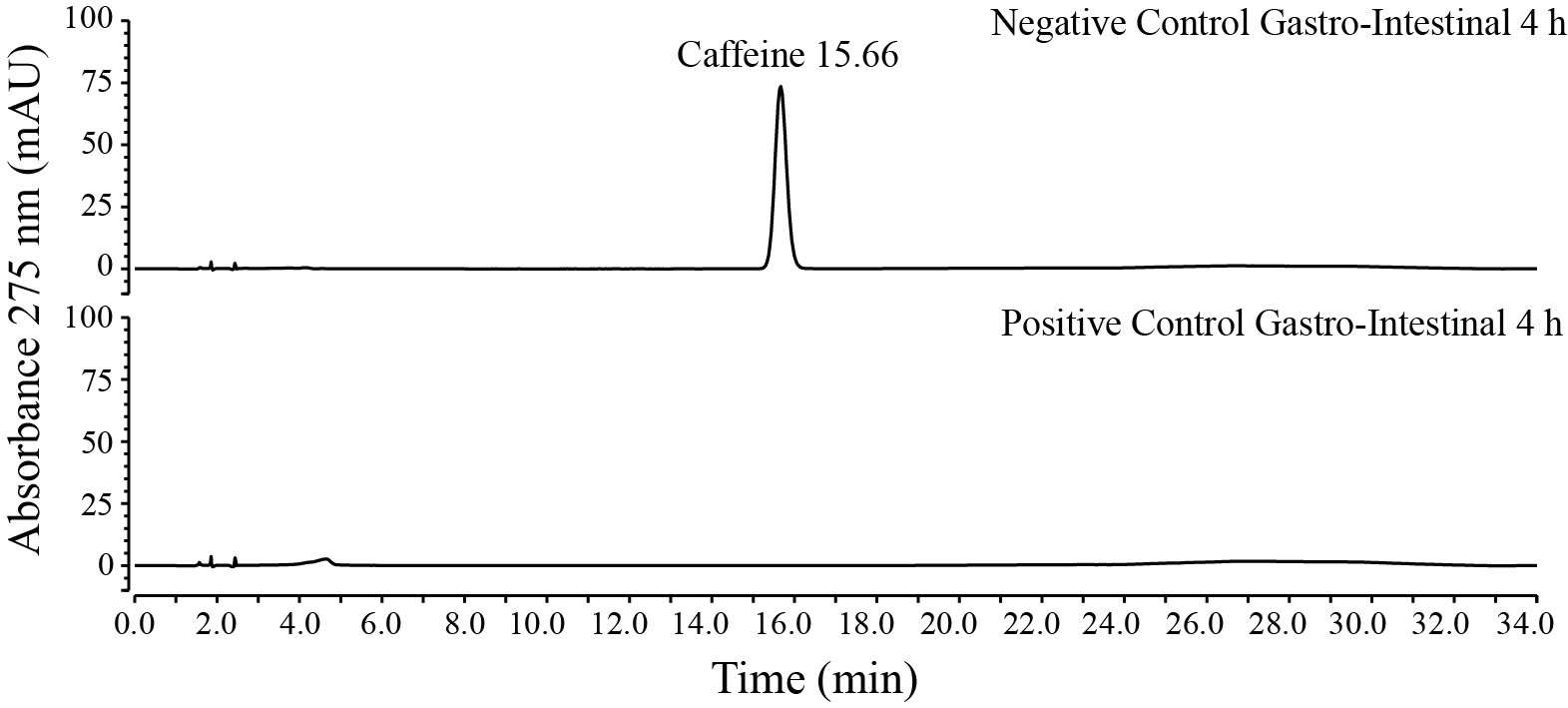


Figure 7S. Comparison of positive and negative controls for gastro-intestinal membrane at 4 hours.


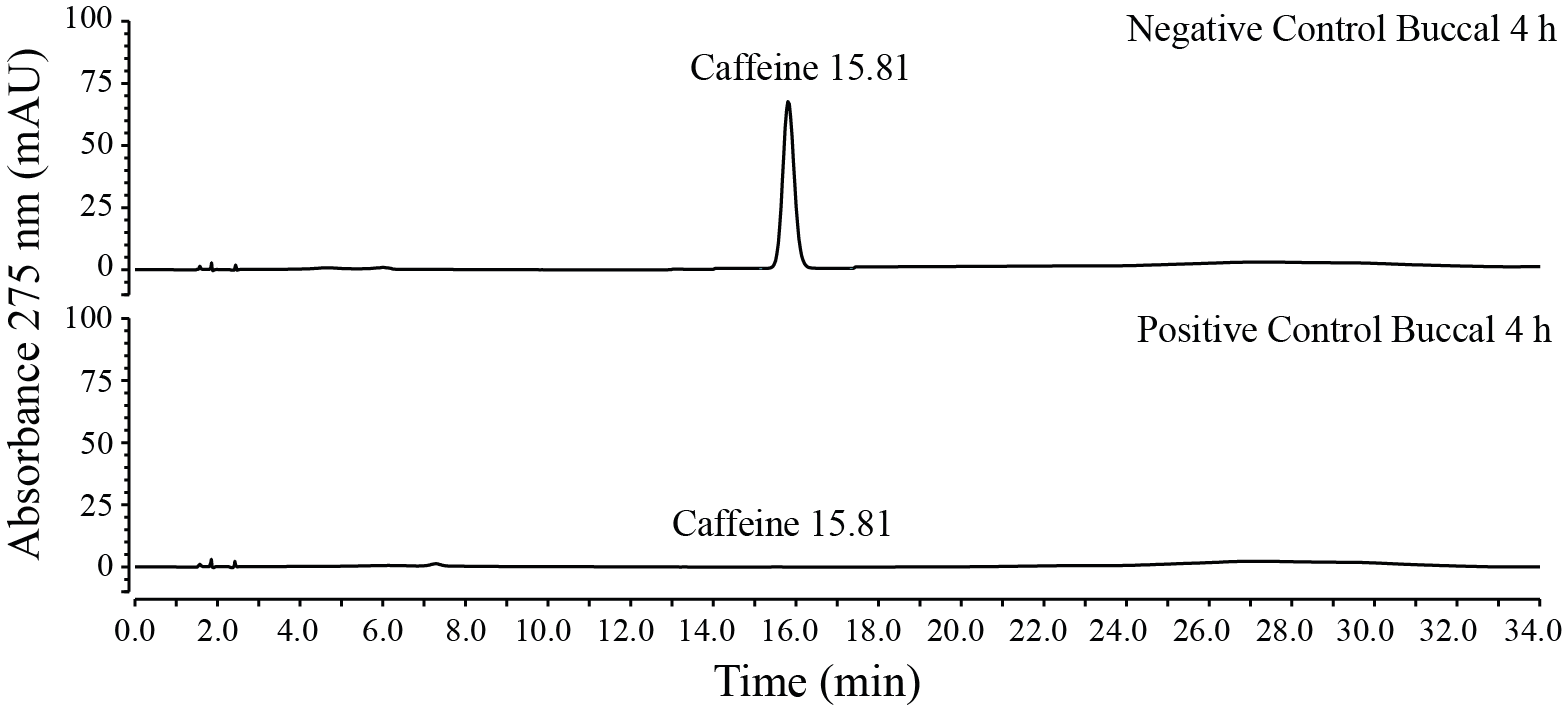


Figure 8S. Comparison of positive and negative controls for buccal membrane at 4 hours.


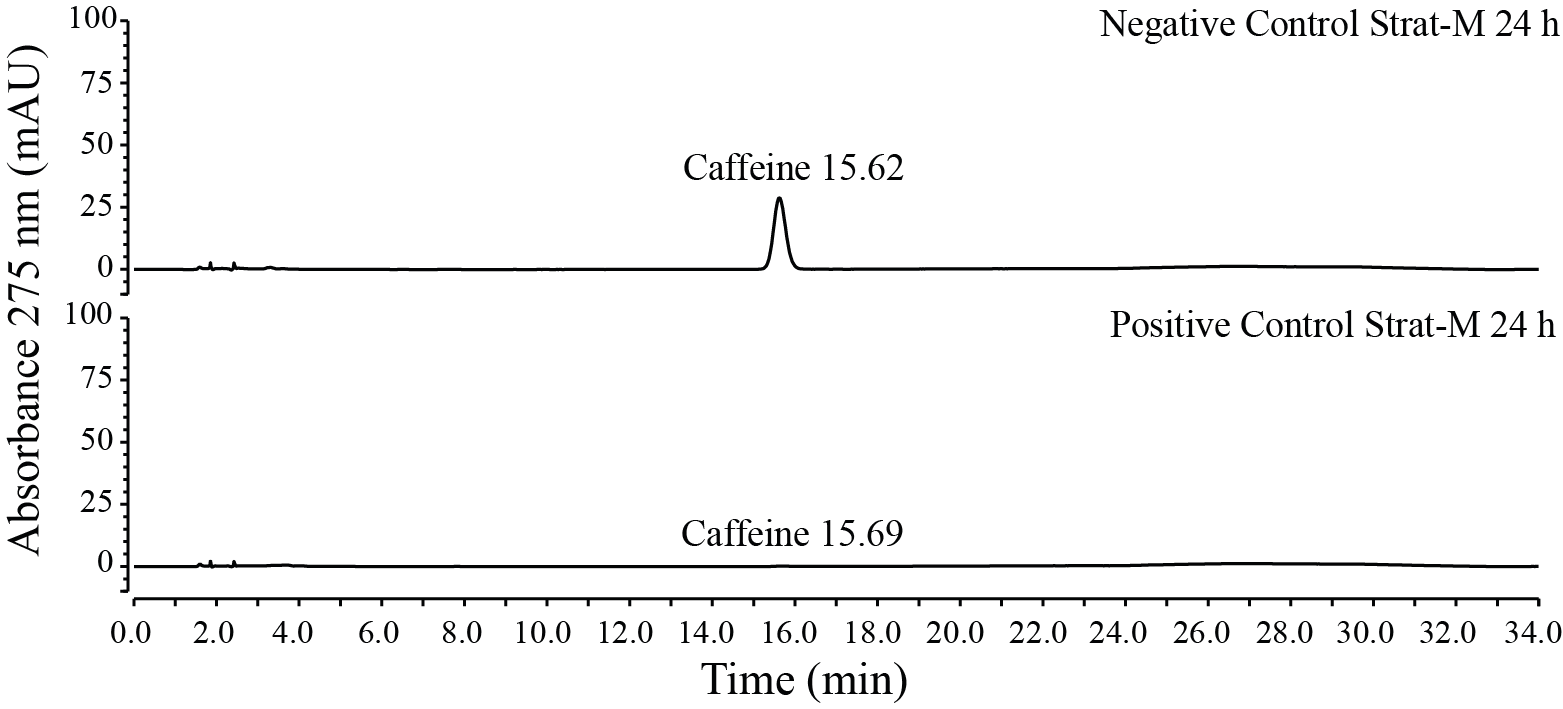


Figure 9S. Comparison of positive and negative controls for STRAT-M® at 24 hours.

**% Barrier effect**

Figure 10S. Barrier effect of SB-MD - Comparison with positive and negative control.

## Supplementary Tables

Table 13S. Mobile phase used for Caffeine HPLC analysis

| **Time (min)** | **Flow (mL/min)** | **% of A: H_2_O** | **% of B: ACN** | **% of C: MeOH** |
| --- | --- | --- | --- | --- |
| 0 | 1.0 | 90 | 7 | 3 |
| 15 | 1.0 | 90 | 7 | 3 |
| 20 | 1.0 | 85 | 10.5 | 4.5 |
| 30 | 1.0 | 85 | 10.5 | 4.5 |
| 35 | 1.0 | 70 | 21 | 9 |
| 40 | 1.0 | 70 | 21 | 9 |
| 45 | 1.0 | 90 | 7 | 3 |
| 50 | 1.0 | 90 | 7 | 3 |

Table 14S. Mobile phase used for Acyclovir HPLC analysis

| **Time (min)** | **Flow (mL/min)** | **% of A: H_2_O** | **% of B: ACN** |
| --- | --- | --- | --- |
| 0 | 1.0 | 98 | 2 |
| 5 | 1.0 | 98 | 2 |
| 27 | 1.0 | 90 | 10 |
| 35 | 1.0 | 80 | 20 |
| 36 | 1.0 | 98 | 2 |
| 40 | 1.0 | 98 | 2 |

Table 15S. Permeability concentration of caffeine (10mg/mL) and acyclovir (1mg/mL) across gastro-intestinal biomimetic membrane.

| **Time** | **Caffeine permeability** | **Acyclovir permeability** |
| --- | --- | --- |
| **(h)** | **µg/mL** | **µg/mL** |
| 0 | 0.000 ± 0.000 | 0.000 ± 0.000 |
| 0.25 | 14.027 ± 9.190 | 0.000 ± 0.000 |
| 0.50 | 50.891 ± 24.536 | 0.000 ± 0.000 |
| 1.00 | 121.464 ± 24.575 | 0.000 ± 0.000 |
| 1.50 | 194.703 ± 18.653 | 1.223 ± 0.009 |
| 2.00 | 242.104 ± 20.727 | 1.491 ± 0.068 |
| 3.00 | 365.280 ± 29.952 | 2.112 ± 0.065 |
| 4.00 | 471.098 ± 32.161 | 2.552 ± 0.082 |

Table 16S. Positive and negative control of caffeine across gastro-intestinal biomimetic membrane.

| **Time** | **Negative control** | **Positive control** |
| --- | --- | --- |
| **(h)** | **µg/mL** | **µg/mL** |
| 0 | 0.000 ± 0.000 | 0.000 ± 0.000 |
| 0.50 | 50.891 ± 24.536 | 0.000 ± 0.000 |
| 1.00 | 121.464 ± 24.575 | 0.000 ± 0.000 |
| 1.50 | 194.703 ± 18.653 | 0.000 ± 0.000 |
| 2.00 | 242.104 ± 20.727 | 0.000 ± 0.000 |
| 3.00 | 365.280 ± 29.952 | 0.000 ± 0.000 |
| 4.00 | 471.098 ± 32.161 | 0.000 ± 0.000 |

Table 17S. Positive and negative control of caffeine across buccal biomimetic membrane.

| **Time** | **Negative control** | **Positive control** |
| --- | --- | --- |
| **(h)** | **µg/mL** | **µg/mL** |
| 0 | 0.000 ± 0.000 | 0.000 ± 0.000 |
| 0.50 | 32.141 ± 14.777 | 0.000 ± 0.000 |
| 1.00 | 86.950 ± 17.426 | 0.000 ± 0.000 |
| 1.50 | 141.555 ± 13.905 | 0.964 ± 1.363 |
| 2.00 | 197.214 ± 17.081 | 2.084 ± 0.033 |
| 3.00 | 280.159 ± 23.092 | 2.119 ± 0.038 |
| 4.00 | 403.751 ± 10.834 | 2.145 ± 0.081 |

Table 18S. Positive and negative control of caffeine across STRAT-M^®^ membrane.

| **Time** | **Negative control** | **Positive control** |
| --- | --- | --- |
| **(h)** | **µg/mL** | **µg/mL** |
| 0 | 0.000 ± 0.000 | 0.000 ± 0.000 |
| 2.00 | 4.344 ± 2.259 | 0.000 ± 0.000 |
| 4.00 | 9.585 ± 0.775 | 0.248 ± 0.429 |
| 6.00 | 14.651 ± 2.504 | 0.770 ± 0.153 |
| 24.00 | 69.241 ± 9.090 | 1.145 ± 0.565 |

1. [] U.S. Departement of Health and Human Service, Food and Drug Administration, Center for Drug Evaluation and Research (CDER), Center for Biologics Evaluation and Research (CBER) (2015). Analytical Procedures and Methods Validation for Drugs and Biologics Guidance for Industry). [↑](#endnote-ref-1)
2. [] Shrivastava, A., Gupta, V., B. (2011). Methods for the determination of limit of detection and limit of quantitation of the analytical methods, 2 (1), 21-25. https://doi.org/10.4103/2229-5186.79345). [↑](#endnote-ref-2)
